# Machine Learning and Artificial Intelligence in Metallic Orthopedic Implant Development: A Narrative Review

**DOI:** 10.3390/ma19143031

**Published:** 2026-07-14

**Authors:** Prajwal Guruprasad, Pranav Sivaram, Andrew Cibik, Pierce T. Bombard, Albert T. Anastasio

**Affiliations:** 1 College of Medicine, Northeast Ohio Medical University, Rootstown, OH 44272, USA; pguruprasad@neomed.edu (P.G.); psivaram@neomed.edu (P.S.); 2 Wake Forest University School of Medicine, Medicine Center Boulevard, Winston-Salem, NC 27157, USA; andrew.cibik@wfusm.edu (A.C.); pierce.bombard@wfusm.edu (P.T.B.); 3 Division of Foot and Ankle, Wake Forest University School of Medicine, Medicine Center Boulevard, Winston-Salem, NC 27157, USA

**Keywords:** machine learning, artificial intelligence, orthopedic implants, titanium alloys, additive manufacturing, lattice structures, surface engineering, corrosion, wear, osseointegration

## Abstract

**Highlights:**

- ML and AI are applied across five stages of metallic orthopedic implant development.
- GAN, Bayesian, and agentic AI platforms accelerate alloy discovery in high-dimensional composition spaces.
- Physics-informed and surrogate ML models optimize AM process parameters and lattice architectures.
- Deep learning enables high-throughput osteogenic screening and exploratory corrosion/wear prediction.
- Clinical translation requires standardized datasets, external validation, and fit-for-purpose regulation.

**Abstract:**

Background: Metallic orthopedic implants face persistent clinical challenges that have proved resistant to incremental conventional development. Machine learning and artificial intelligence offer a complementary paradigm for navigating the high-dimensional design spaces governing implant performance, yet the literature remains fragmented across disciplinary silos with no comprehensive synthesis spanning the full development pipeline. Methods: A structured database search of PubMed/MEDLINE, Embase, and Cochrane (executed May 2026), supplemented by hand-searching of reference lists, identified 33 primary studies organized across five sequential domains: alloy composition discovery, additive manufacturing process–property optimization, lattice and porous structure design, surface engineering and coatings, and corrosion and wear prediction. Results: Across all five domains, machine learning approaches, including random forests, convolutional neural networks, Bayesian optimization, generative adversarial networks, physics-informed neural networks, and autonomous multi-agent platforms, have accelerated property prediction and design space exploration beyond experimental or simulation-based methods. Shared barriers to translation include small, heterogeneous datasets, reliance on internal rather than external validation, limited interpretability, and the absence of regulatory frameworks for AI-assisted device design. Representative performance included modulus predictions within ~4 GPa of first-principles values, ML-designed alloys reaching ~42.7 GPa (versus 103–120 GPa for Ti-6Al-4V), property prediction R^2^ often above 0.90 (up to 0.96–0.9991), 98.3% corrosion severity classification accuracy, and acceleration from a roughly fivefold reduction in finite element simulations to surrogates compressing days into minutes. Conclusions: Addressing these limitations will require open standardized databases linking materials parameters to registry-level clinical outcomes, prospective clinical validation studies, and coordinated engagement between researchers, industry, and regulatory agencies.

## 1. Introduction

Metallic implants, chiefly titanium (Ti) alloys, cobalt–chromium (CoCr) alloys, and stainless steel, have underpinned joint replacement and fracture fixation for over half a century [1,2]. Despite decades of refinement, several persistent problems still limit long-term survivorship. Stress shielding arises because metallic implants are far stiffer than bone (the modulus of Ti-6Al-4V [103–120 GPa] greatly exceeds cortical bone [7–25 GPa]), redirecting load and driving bone resorption and failure [3,4]. The principal consequence is aseptic loosening, which accounts for ~31% of hip revisions and is a leading indication for late knee revision [5,6]. Corrosion and tribocorrosion at modular junctions release ions and debris that drive macrophage-mediated inflammation and osteolysis, while wear particles amplify bone resorption via a TNF-α-driven osteoclast cascade [7,8,9]. Implant-associated biofilm infection compounds these risks: once established, biofilms resist antibiotics and host immunity, and surgical strategies often fail [10,11]. Addressing these challenges demands optimization across length scales, from alloy electronic structure to whole-joint kinematics, a task that has outpaced conventional approaches [12].

Machine learning (ML) and artificial intelligence (AI) offer a different paradigm for these high-dimensional design spaces. Rather than first-principles simulation or exhaustive trial and error, ML extracts patterns from existing data, builds surrogate models at a fraction of measurement cost, and drives active-learning loops that locate optima efficiently [13]. Broad reviews have begun to chronicle this shift: Zhou et al. surveyed AI-assisted biomaterial design and property prediction, De Wei et al. synthesized ML across the full biomaterials R&D pipeline, and Suwardi et al. and Basu et al. traced data-driven paradigm shifts and formalized “biomaterialomics” across material classes [12,14,15,16]. Collectively, they identify data scarcity, interpretability, and regulatory readiness as dominant barriers to translation.

Nevertheless, this literature remains siloed: alloy discovery ML rarely engages with additive manufacturing (AM) process control, lattice researchers seldom consult the surface engineering literature on osseointegration, and corrosion or wear models have evolved in isolation from upstream design [17,18,19,20,21,22]. No comprehensive review has yet synthesized ML and AI progress across all of these interconnected domains for metallic orthopedic implants. In contrast to existing biomaterials reviews that survey ML across polymeric, ceramic, and metallic systems at a general level [12,14,15,16], the distinct contribution of this review is its exclusive focus on the metallic-implant pipeline and the materials-specific phenomena that govern it, including alloy chemistry and β-phase stability, additive manufacturing defect formation, fatigue behavior, tribocorrosion, and long-term implant survivorship, none of which is adequately captured by cross-material syntheses.

This review organizes the literature into five sequential domains: (1) alloy composition discovery (mechanical properties, phase stability, biocompatibility); (2) AM process–property optimization (porosity, microstructure, and fatigue from LPBF parameters); (3) lattice and porous structure design (generative, topology-optimization, Bayesian, and surrogate approaches); (4) surface engineering and coatings (osteogenic response and antibacterial textures); and (5) corrosion and wear prediction (tribocorrosion, wear, and imaging-based failure detection). It closes by identifying barriers to clinical translation and proposing a roadmap for integrated, multi-domain ML pipelines linking computational prediction to patient outcomes.

## 2. Methods

This narrative review was conducted following established best practices for literature synthesis in biomedical materials science. A structured search was performed across three electronic databases: PubMed/MEDLINE, Embase, and Cochrane. Searches were executed in May 2026, capturing ML and AI development in the orthopedic materials field. No language restrictions were applied; however, all included studies were available in English.

The search strategy used was as follows: (“machine learning” OR “deep learning” OR “artificial intelligence” OR “neural network” OR “random forest” OR “Bayesian optimization” OR “generative design” OR “CALPHAD”) AND (“titanium alloy” OR “cobalt-chromium” OR “stainless steel” OR “metallic implant” OR “Ti-6Al-4V” OR “lattice structure” OR “additive manufacturing” OR “surface coating” OR “corrosion” OR “wear prediction”) AND (“orthopedic” OR “orthopaedic” OR “bone” OR “implant”). Search results were supplemented by a set of preselected articles known to be relevant based on prior knowledge. Reference lists of retrieved reviews and key primary studies were hand-searched for additional relevant publications.

Two reviewers independently screened 983 titles and abstracts for relevance to metallic orthopedic implants and ML/AI. Studies were included if they (1) used ML/AI as a primary analytical tool, (2) targeted an orthopedic-relevant metallic system (titanium, cobalt-chromium, stainless steel, high-entropy, or biodegradable magnesium alloys), (3) addressed at least one of the five domains, and (4) were original research or systematic reviews. Studies were excluded if they were purely polymeric/ceramic, applied ML only to clinical outcomes without materials-level analysis, or lacked methodological detail; conference abstracts, editorials, and opinion pieces were also excluded.

Following screening, 33 primary studies were identified and categorized across five thematic domains that map onto sequential stages of the implant development pipeline: (1) alloy composition discovery, (2) AM process–property optimization, (3) lattice and porous structure design, (4) surface engineering and coatings, and (5) corrosion and wear prediction. Data were extracted narratively, with emphasis on ML architecture employed, dataset characteristics (size, source, material system), performance metrics reported, and degree of experimental validation. Given the heterogeneity of study designs, quantitative analysis was not performed.

**Figure 1:**
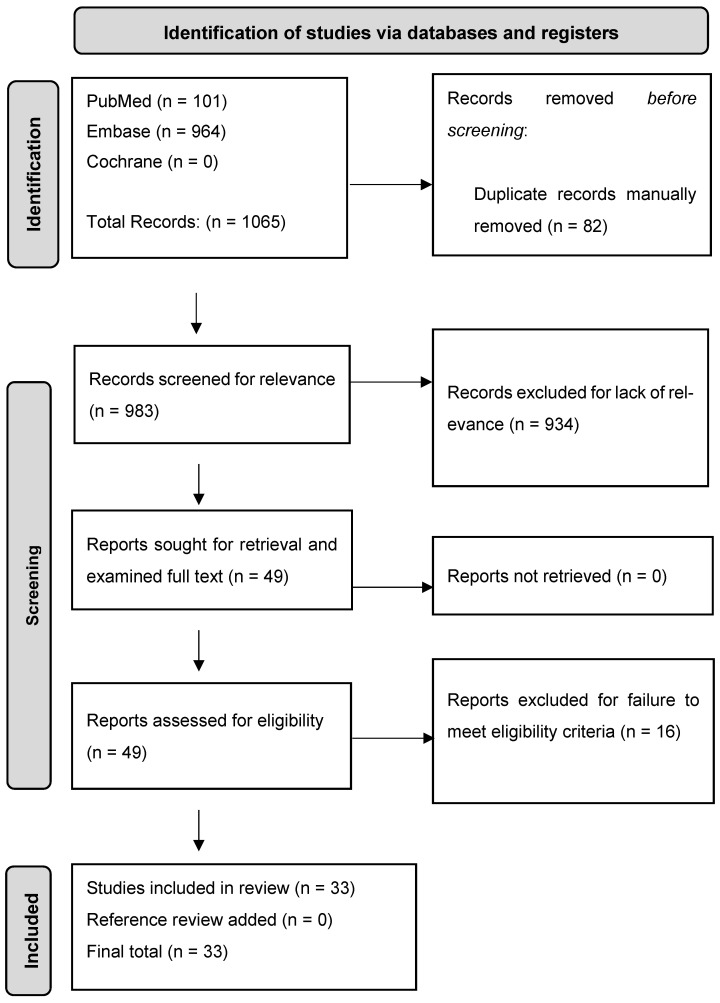
PRISMA-style flow of study identification, screening, and inclusion. Duplicate records were removed by automated title/author/DOI matching with manual verification prior to screening. Intermediate duplicate and full-text exclusion sub-counts should be confirmed against the authors’ screening log.

Records were exported to a reference manager and de-duplicated by automated title/author/DOI matching with manual verification. Two reviewers independently performed title/abstract and full-text screening; disagreements over inclusion, domain, or scope were resolved by discussion or senior-author adjudication. Each study was assigned to one primary domain by its principal contribution, with multi-domain studies cross-referenced. The selection process is summarized in Figure 1 and the included studies in Table 1.

PubMed/MEDLINE, Embase, and Cochrane were chosen to prioritize biomedical and clinically indexed literature relevant to orthopedic implant application. This biomedical focus, together with deliberately stringent inclusion criteria requiring both a metallic implant-relevant material system and a primary machine-learning analytical contribution, accounts for the comparatively small final yield of 33 studies from 983 screened records. We acknowledge that not searching engineering-indexed databases such as Scopus, Web of Science, IEEE Xplore, and Engineering Village is a limitation that may have excluded relevant materials-science and computational reports indexed outside the biomedical literature; this is revisited in the Section 8.

## 3. Alloy Composition Discovery

The selection of alloy composition is the first and most consequential decision in implant design, as it determines the fundamental mechanical, corrosion, and biological envelope within which all downstream engineering must operate. The ideal biomedical metallic alloy combines a low elastic modulus matching cortical bone (10–30 GPa) to minimize stress shielding, high strength and ductility, excellent corrosion resistance, and freedom from cytotoxic elements such as aluminum and vanadium [51]. The compositional space of multicomponent metallic systems is astronomically large, rendering exhaustive experimental exploration infeasible [52]. ML approaches have therefore attracted considerable attention as tools to accelerate this search.

### 3.1. Regression-Based Property Prediction

Early ML efforts focused on regression models trained on curated experimental databases. Marković et al. constructed a database of 246 biocompatible titanium alloys encompassing composition, Young’s modulus, and thermal properties, then trained an Extra Tree Regression model to predict elastic modulus [23]. Their analysis identified specific heat as the single most influential descriptor for lowering Young’s modulus, a non-intuitive insight that would have been difficult to extract by conventional metallurgical reasoning alone. A Monte Carlo simulation of ten million hypothetical compositions, constrained to produce moduli below 70 GPa, identified multicomponent Ti-Zr-Sn-Mn-Nb alloys as experimentally actionable targets.

More sophisticated physics-informed approaches also followed. Salvador et al. trained linear-regression, random-forest, and neural-network models on elasticity data from over 1800 Materials Project compounds to predict bulk and shear moduli across the Ti-Nb-Zr system at 2 at.% (atomic percent) increments [18]. Random forests proved most reliable, deviating from neural network results by less than 4 GPa. Density functional theory (DFT) verification confirmed that ~22 at.% Zr gave low moduli with favorable beta-phase stability, and that Nb above 14.8 at.% suppressed the high-modulus omega phase. This pairing of data-driven screening with physics-based validation exemplifies an increasingly favored hybrid strategy.

The ambition of these approaches has grown accordingly. Su et al. built an ML framework for laser powder bed fusion (LPBF) integrating beta-phase stability, modulus, printability, and biocompatibility to identify a Ti-Nb-Ta-Zr-Sn composition, later fabricated and experimentally validated [17]. The alloy showed a Young’s modulus of ~42.7 GPa and ~30.9% ductility from a metastable beta-phase microstructure with cubic texture and reduced dislocation density, outperforming commercial Ti-6Al-4V for stress shielding mitigation. Solano-Orrala et al. reviewed the clinical promise of metastable β-Ti alloys, highlighting ML-assisted composition screening as a key enabler for the vast β-stabilizer design space [53].

### 3.2. Generative and Inverse Design

A persistent challenge is data scarcity: experimental alloy databases are modest and biased toward previously studied compositions. Zhang et al. addressed this with an algorithm combining generative adversarial networks (GANs) [54] and proximal policy optimization (PPO) [24]. The GAN generates tens of thousands of synthetic samples from only hundreds of experimental points, expanding the training landscape. PPO then reframes composition design as a Markov decision process, improving search through agent-environment interaction. Versus traditional optimization, the approach improved both efficiency and accuracy, offering a scalable route to reverse-design complex alloys.

The reach of ML-driven alloy design also extends beyond titanium to high-entropy alloys (HEAs), equimolar or near-equimolar multicomponent systems whose vast compositional freedom can yield potentially superior combinations of strength, corrosion resistance, and biocompatibility relevant to next-generation implants. Gupta et al. deployed cross-property deep transfer learning models that leverage source models trained on large datasets to build predictive models for small-dataset target properties, ultimately predicting up to 41 distinct material property values from composition alone via an online tool. This transfer learning strategy is particularly valuable for HEAs, where experimental databases remain sparse relative to the enormous compositional landscape [25,26].

### 3.3. Autonomous Multi-Agent Platforms

The most ambitious recent contribution is the AtomAgents platform of Ghafarollahi and Buehler, a physics-aware generative framework in which multiple large-language-model (LLM) agents collaborate autonomously by retrieving domain knowledge, running atomistic simulations, and analyzing results to address complex alloy-design tasks [27]. It predicted properties accurately across alloy systems and highlighted solid-solution alloying in steering property profiles, without human orchestration of the workflow. This agentic paradigm is a potential step-change: the system itself reasons about which tools are needed, executes them, and synthesizes findings. Whether such systems can be robustly validated and regulated for safety-critical medical device applications remains an important open question.

Collectively, these studies show an evolution from single-property regression on small datasets toward multi-property, physics-constrained, increasingly autonomous platforms. Two overarching challenges remain. The first is data: alloy databases are heterogeneous and biased toward previously studied compositions, so open, standardized property repositories (analogous to the Materials Project) will be essential [55]. The second is scope: as Rabbitt et al. argued, biological criteria (immune response, angiogenesis, osseointegration, and infection resistance) should enter alloy design from the outset rather than repurposing alloys from other industries [56]. ML is increasingly adept at linking alloying elements to mechanical properties, but composition–bioresponse correlations remain poorly understood. Closing that gap, and establishing whether autonomous platforms can be validated for safety-critical use, will define the next phase.

A recurring methodological consideration across these alloy-design studies is the choice of input descriptors. Looking forward, alloy-design models should prioritize descriptors grounded in alloy theory rather than composition alone, such as β-phase stability and molybdenum equivalent, valence electron concentration, atomic size mismatch, electronegativity difference, and computed elastic constants, together with descriptors of corrosion tendency and the cytotoxicity of individual alloying elements such as aluminum and vanadium. Descriptors of this kind are expected to extrapolate more reliably beyond the training distribution than raw elemental fractions, and to yield feature-importance rankings more readily reconciled with established metallurgical mechanisms.

### 3.4. Critique

Despite maturation toward physics-constrained and generative approaches, weaknesses recur. Reported accuracies rest on R^2^ and error metrics from held-out subsets of small, composition-biased databases, with little reporting of overfitting controls, learning curves, or data-leakage safeguards against near-duplicate compositions split across train and test sets. Generative strategies such as GAN–PPO [24] expand sparse data but risk reproducing seed-data biases rather than genuine diversity, so outputs require experimental or first-principles confirmation before being treated as discoveries. Clinically, the design target is a modulus approaching cortical bone (≈10–30 GPa); reductions from 103–120 GPa toward the demonstrated ~42.7 GPa [17] are meaningful for stress-shielding mitigation, whereas sub-GPa precision is immaterial given measurement scatter. No reviewed model is yet accurate enough to displace confirmatory mechanical and biological testing; ML here is best positioned to prioritize candidates for experiment, not replace it.

## 4. Additive Manufacturing Process–Property Optimization

AM, particularly LPBF and electron beam melting (EBM), has transformed the production of metallic orthopedic implants by enabling geometrically complex, patient-specific architectures impossible to fabricate by conventional subtractive methods [57]. However, AM introduces a process parameter space of high dimensionality: laser power, scan speed, hatch spacing, layer thickness, scan strategy, build orientation, and island size interact nonlinearly to determine porosity distribution, microstructure, residual stress, and ultimately mechanical properties of the finished part [58]. Empirical process optimization by design of experiments is time-consuming and costly; ML offers a path to more efficient process mapping and real-time quality control [59].

### 4.1. Property and Defect Prediction

The relationship between volumetric energy density (VED) and part quality has been a central focus of early ML work. Hassanin et al. introduced a deep learning neural network (DLNN) to predict densification and hardness of LPBF-fabricated Ti-6Al-2Sn-4Zr-6Mo, a high-strength alpha-beta alloy with no prior LPBF processing history [28]. The DLNN achieved mean percentage errors of 3% for porosity and 0.2% for hardness, enabling generation of process maps identifying a near-full densification window at 77–113 J/mm^3^ and revealing that hardness increased with energy density while remaining sensitive to island size at high VED. Notably, this performance was achieved with small training datasets, a practically important finding given the cost of AM experimental campaigns.

The critical role of surface roughness and pore characteristics in fatigue performance was systematically quantified by Moon et al., who printed 197 fatigue bars using the same laser power but varied scanning speeds to produce a range of pore geometries characterized by micro-computed tomography [29]. A dropout neural network (DONN) was employed to link surface and pore features to fatigue life (logN), demonstrating good prediction accuracy while also providing uncertainty estimates for each prediction. For machined samples, in which surface pores resemble internal pores, fatigue life was highly correlated with average pore size and projected pore area in the plane perpendicular to the stress direction, yielding physically interpretable design guidance alongside ML predictions.

Hossain et al. introduced a hybrid multimodal surrogate for LPBF property prediction, coupling engineered process–physics features with morphology proxies via a two-stage embedding module and gradient-boosted tree regressors [30]. On small, set-level datasets with intermittently missing descriptors, it achieved RMSE/R^2^ of 11.07 MPa/0.895 (yield strength), 13.88 MPa/0.873 (ultimate tensile strength), and 0.677%/0.861 (elongation) across six targets. Surface roughness and hardness proved more challenging (R^2^ ≈ 0.11–0.12), reflecting the complexity of surface formation mechanisms. It also enables constraint-aware candidate generation, identifying recipes that balance strength and surface objectives under uncertainty: a blueprint for multimodal, small-data AM.

Convolutional neural networks (CNNs) have linked microstructure to properties. Zhang et al.’s Metallography–Property Relationship Neural Network (MPR-Net) predicts tensile strength and Vickers hardness from optical metallography of LPBF 316L stainless steel, achieving R^2^ of 0.96 and 0.91 [31]. Grad-CAM and clustering revealed that the network weighted melt-pool morphology and grain patterns most heavily, bridging statistical prediction and physical interpretation. Pan et al. applied a DeepLabV3+ segmentation network to synchrotron computed-tomography data of LPBF Ti-6Al-4V, reaching 98.2% pixel accuracy for defect segmentation [32]. Segmented defects then predicted fatigue lifetime within a ±2.2 scatter band, linking automated defect characterization to structural-integrity prediction.

### 4.2. Physics-Informed and Integrated Models

Physics-informed approaches represent an important complementary strategy. Wang et al. developed a defect-driven physics-informed neural network (PINN) that embeds critical defect information directly into the loss function during training, enabling the model to capture physical constraints without explicit finite element modeling [33]. Under small-sample conditions representative of AM experimental campaigns, the defect-driven PINN outperformed both pure data-driven models and fracture-mechanics-based PINNs in generalization accuracy, providing a physically consistent and scalable framework for fatigue life prediction across multiple AM material systems.

The most clinically integrated AM-ML framework to date, from Zaheer et al., combined LPBF fabrication of Ti-6Al-4V, finite-element (FE) simulation of fracture healing, and AI regression into a unified pipeline for implant evaluation [34]. Models trained on mechanobiological FE outcomes predicted healing trajectories (deviatoric strain, fluid velocity, and pore pressure) in real time without repeated FE runs. Such surrogates could accelerate patient-specific implant design. Sanchez et al. extended process–property ML to creep in LPBF Alloy 718, with ensemble feature importance analysis identifying part density, pore count, build orientation, and scan strategy as dominant determinants of minimum creep rate, predicted to within 1.40% [35].

### 4.3. Defect Physics and Model Transferability

A recurring challenge is the limited transferability of ML models across material systems, machine platforms, and laboratories. Developing physically informed, transferable process–property models, potentially through PINNs that embed conservation equations as constraints, is an active research priority [59,60]. In situ process monitoring data streams (pyrometry, optical emission spectroscopy, X-ray tomography) represent a largely untapped opportunity to build real-time defect detection systems that could close the loop between ML prediction and AM process control [61].

Across these AM studies, inputs and outputs map onto fatigue-critical defect-physics variables: laser power, scan speed, hatch spacing, and volumetric energy density govern lack-of-fusion porosity (low energy) versus keyhole porosity (high energy), while build orientation, layer thickness, and scan strategy shape residual stress, roughness, and melt-pool morphology [58,59]. Post-processing, including heat treatment and hot isostatic pressing, further modifies pore populations and residual stress and remains an underexplored model input. Linking these specific descriptors, rather than energy density alone, to fatigue outputs is what lets the dropout and defect-driven PINN approaches yield physically interpretable predictions.

### 4.4. Critique

The AM literature spans three modeling paradigms of which the merits depend on data availability. Purely data-driven models perform well within their training process window but transfer poorly across machines, feedstocks, and laboratories. Physics-informed approaches such as the defect-driven PINN [33] are generally expected to generalize better than purely data-driven models under the small samples typical of AM, with data-driven architectures becoming competitive as datasets grow. It also shows where ML fails: the same multimodal surrogate that predicted yield strength and elongation well (R^2^ ≈ 0.87–0.90) reached only R^2^ ≈ 0.11–0.12 for surface roughness and hardness [30], a reminder that aggregate performance can mask domain-specific failure. Physics-informed and transfer-learning strategies, validated across platforms rather than within-dataset hold-outs, should be prioritized.

## 5. Lattice and Porous Structure Design

Porous architectures work through two mechanisms: lowering the construct’s effective modulus to match bone (mitigating stress shielding) and providing interconnected pores for vascular ingrowth and osseointegration [62]. Their design requires simultaneous optimization of pore size, strut geometry, porosity, gradients, and anisotropy: a challenge well suited to ML and generative design [62,63]. Minku and Ghosh studied total ankle replacement (TAR), where poor implant-bone fixation from aseptic loosening remains a dominant challenge. Their first study designed four porous diamond-structured tibial implants (PDSTI) at 50–80% porosity, evaluated with macro-microscale FE modeling and a physics-based mechanoregulatory tissue differentiation algorithm [36]. ML models trained on the FE data predicted site-specific bone ingrowth, enabling rapid porosity screening. The 70% configuration was optimal, showing superior bone formation and load transfer versus a solid implant, while 80% was detrimental, showing that maximizing porosity is counterproductive. A follow-on study extended the framework to porous rhombic dodecahedron tibial implants (PRDTIs), training four artificial neural network (ANN) models for faster bone-ingrowth prediction. PRDTI70 again maximized bone formation while raising tibial von Mises stresses versus lower-porosity and solid constructs, reinforcing that each geometry has its own porosity sweet spot [19].

### 5.1. Surrogate Modeling of Lattice Mechanics

Generative and surrogate modeling approaches have been applied to strut-geometry optimization within lattice unit cells. Lee et al. investigated beam element shapes using a deep learning approach parameterized by high-order Bézier curves, combined with a hybrid neural network and genetic optimization (NN-GO) method [37]. By shifting material distribution toward joint regions, which are the mechanically weakest locations, the optimized designs balanced axial and bending deformation modes to achieve the highest modulus and strength among benchmarked configurations. LPBF fabrication and compression testing validated the computational predictions, confirming that smoother strut transitions enabled by high-order Bézier parameterization reduced stress concentrations and improved both stiffness and energy absorption.

The challenge of efficiently navigating high-dimensional lattice design spaces motivated the active learning approach of Gongora et al., who combined ML surrogate modeling with Shapley additive explanation (SHAP) analysis and Bayesian optimization [38]. Their ML surrogate models achieved prediction R^2^ values above 0.95, and SHAP analysis identified which design variables most influenced mechanical performance, providing mechanistic understanding alongside optimization. Bayesian optimization reduced the required number of FE simulations by approximately fivefold compared to grid-based search, demonstrating the substantial computational savings achievable through active learning.

Gyroid lattice structures (GLS) attract interest for near-isotropy and tunable porosity. Judawisastra et al. ran FE analysis of GLS across relative densities of 10–75%, experimentally validated predictions, and built a Gaussian Process Regression (GPR) surrogate for relative elastic moduli across the full density range [39]. The GPR model substantially outperformed the classical Gibson-Ashby model at high relative densities, where Gibson-Ashby systematically diverges. Shetty et al. extended this to 3D-printed PLA+ scaffolds with Lidinoid, Diamond, and Gyroid geometries at 1.0–2.0 mm wall thickness [40]. A back-propagation ANN predicted displacement and strain (R^2^ = 0.9991 and 0.9954), with Gyroid showing the best mechanical integrity (least displacement 0.36 mm and strain 1.2 × 10^−2^ at 3 kN, 2.0 mm thickness).

Jana et al. addressed a clinically novel challenge: predicting the effective Young’s modulus of titanium scaffolds partially filled with biodegradable magnesium as a function of scaffold geometry and relative density [41]. As the Mg-filled portions dissolve with bone ingrowth, the effective stiffness of the construct changes over time; an AI model trained on numerical homogenization data across 18 geometries predicted the Ti/Mg ratio required to achieve target stiffness for specific patient scenarios. This time-evolving stiffness-matching concept, in which the implant progressively transfers load to the regenerating bone, represents a promising direction for next-generation degradable composite scaffolds.

### 5.2. Patient-Specific and Multifunctional Design

An important frontier is the integration of patient-specific loading data with lattice optimization. Current studies typically optimize against population-average loading conditions; in reality, the stress environment experienced by a given implant depends on patient body mass, gait pattern, activity level, and bone quality [64]. Connecting ML-optimized lattice architectures to patient-specific musculoskeletal models and FE simulations could yield implants tailored not merely to anatomy but to the individual biomechanical context of each patient, representing a form of truly personalized implant engineering [65,66,67].

Although one of these studies [40] employed polymeric PLA+ scaffolds rather than a metallic system, it is retained here because the triply periodic minimal-surface geometries and the surrogate modeling methodology it evaluates are directly transferable to metallic lattice fabrication; its inclusion is methodological rather than material. More broadly, porosity and effective modulus alone are insufficient design targets. Clinically meaningful lattice optimization must jointly account for pore interconnectivity and permeability, which govern vascular and bone ingrowth, architectural anisotropy, strut thickness and junction geometry, which control local stress concentration and fatigue strength, and the resulting fatigue life under physiological cyclic loading, dimensions that the reviewed ML surrogates capture only partially [62,63].

### 5.3. Critique

Most lattice studies train surrogate models on finite-element-generated datasets and validate against further simulations or a small number of printed specimens, so predictive accuracy is reported against the FE ground truth rather than against in vivo bone ingrowth. The recurring finding that an intermediate porosity (≈70%) is optimal while higher porosity (80%) is detrimental [19,36] is a valuable negative result, demonstrating that naive maximization of porosity degrades performance, but it also exposes that current models optimize against mechanobiological surrogates whose own fidelity to clinical osseointegration is unverified. Bayesian and active learning approaches offer the clearest efficiency gains, an approximately fivefold reduction in required simulations [38], and should be favored where each evaluation is computationally expensive. On the question of which paradigm suits which data scale, Gaussian-process regression and Bayesian or active-learning methods are most efficient [39] in the low-data regime typical of finite-element-generated lattice datasets, using their uncertainty estimates to guide sampling, whereas artificial neural networks become competitive only once larger simulation-generated datasets are available [40].

## 6. Surface Engineering and Coatings

The interface between implant surface and host biology is the proximate determinant of osseointegration quality, infection risk, and long-term fixation stability [68,69]. Surface modification strategies for titanium and other metallic implants encompass acid etching, sandblasting, anodization, plasma spraying, laser texturing, and chemical coatings, each producing distinct topographies, chemistries, and wettability profiles that influence cell adhesion, osteogenic differentiation, and bacterial colonization [70]. The multiplicity of interacting surface parameters, combined with the cost and throughput limitations of biological assays, makes surface optimization a natural application for ML [13,16,44].

### 6.1. Osteogenic and Cell-Response Prediction

Li et al. addressed the throughput bottleneck by developing the Orthopedic Implants-Osteogenic Differentiation Network (OIODNet), a deep learning model trained on early cell morphology images paired with alkaline phosphatase (ALP) activity values from cells cultured on a range of titanium and alloy surfaces [20]. OIODNet achieved performance metrics exceeding 0.98 across all six evaluation parameters, and its predictions were validated against ALP outcomes for metal-polyphenol network (MPN) coatings. The Osteogenic Predictor application packages this capability into an accessible tool, potentially transforming a week-long differentiation assay into a same-day computational prediction and enabling high-throughput screening of novel surface modifications.

Lu et al. modeled the nonlinear relationship between micro/nano-surface structures and femtosecond laser parameters with a GA-BP (Genetic Algorithm–Back Propagation) neural network trained on 256 data points, then designed titanium surfaces decorated with silver nanoparticles (AgNPs) [42]. The network achieved sub-10% errors for laser-induced periodic surface-structure dimensions, enabling control of topography, wettability, and Ag^+^ release. The resulting hydrophobic surfaces prevented Ag^+^ burst release while sustaining antibacterial efficacy against Gram-negative (*E. coli*, 89.27%, MCs1) and Gram-positive (*S. aureus*, 65.4%, LIPSS) bacteria, and remaining cytocompatible with murine preosteoblasts (MC3T3-E1). Reconciling competing antibacterial and osseointegration demands through ML-informed surface design is clinically important, since infection and poor fixation are both major drivers of revision.

Ghosh et al. employed a back-propagation neural network (BPNN) in conjunction with FE simulation and genetic algorithm search to optimize implant surface macro-textures for maximal bone growth subject to clinically admissible micromotion constraints [43]. The BPNN trained on FE-predicted datasets enabled the genetic algorithm to search efficiently across the texture design space, identifying optimal osseointegration-maximized textures (OMTs) featuring periodic rib patterns with alternating dimensions that provide a uniform stress environment at the bone-implant interface. The results revealed that certain reductions in rib and groove dimensions promote bone growth, a finding that would require prohibitively large FE campaigns to obtain without ML surrogates.

### 6.2. Multifunctional Surfaces and Coatings

Fang et al. introduced a high-throughput rational design strategy combining gradient surface generation with statistical learning for multifunctional orthopedic implant surfaces [44]. Rather than optimizing for a single biological activity, the approach simultaneously screened osteogenic, angiogenic, and neurogenic performance across a ternary functionalized surface design space. Statistical learning models not only screened parameter combinations in high-throughput fashion but also extrapolated optimal conditions beyond the experimentally tested range. The resulting ternary functionalized surface was validated in vitro and in vivo, confirming optimal osseointegration promotion and demonstrating the feasibility of ML-guided rational design for multimodal biological surfaces.

Two studies advanced ML-guided coatings for biodegradable magnesium alloys. Barai et al. combined Response Surface Methodology (RSM) with Central Composite Design, ANN modeling, and multi-objective optimization (NSGA-II, TLBO, MOPSO) for a stearic acid/ZnCl_2_ coating on Mg alloys [45]. After augmentation, the ANN reached R^2^ > 0.99, and the NSGA-II Pareto front yielded a superhydrophobic surface (contact angle 152° ± 1°), 92.4% corrosion resistance efficiency, and a 0.180 mm/year corrosion rate. Live/dead cell assays confirmed biocompatibility with increased cell proliferation at 48 h. Khakbiz and Esteghza used Gaussian Process Regression (GPR) to predict surface-topography parameters of plasma-electrolytic-oxidation (PEO)-coated Mg-5.5Zn-1.5Si, closely matching experimental SPIP™ measurements of roughness, waviness, and isotropy [46]. PEO coatings raised surface hardness six-fold (81 HV → 480 HV) and cut corrosion current density (64.56 µA/cm^2^ → 0.72 µA/cm^2^), with GPR providing a predictive tool for surface optimization across biodegradable-implant pipelines.

A cross-cutting challenge in surface engineering ML is the limited correlation between in vitro biological markers and in vivo osseointegration outcomes [71]. Building ML models with genuine predictive validity for clinical performance will ultimately require training on datasets linking detailed surface characterization to in vivo and registry-level outcomes, a data integration challenge of considerable technical and logistical complexity that has not yet been addressed in the literature reviewed here.

### 6.3. Critique

Surface-engineering ML has produced the strongest in vitro performance metrics in this review, with several models exceeding an R^2^ or accuracy of 0.98 [20,45], but these are predominantly trained on short-term in vitro readouts, principally cell morphology, alkaline phosphatase activity, and antibacterial efficacy, that are imperfect proxies for clinically meaningful endpoints such as long-term fixation, immune tolerance, and infection resistance. High in vitro predictive accuracy should therefore not be conflated with clinical predictive validity. The few studies incorporating in vivo validation, for example, the ternary functionalized surface [44], are the more clinically credible, and the principal limitation across the subfield remains the near-absence of datasets linking quantitative surface characterization to in vivo and registry-level outcomes.

## 7. Corrosion and Wear Prediction

The in-service degradation of orthopedic implants through corrosion, wear, and their synergistic combination (tribocorrosion) is a leading cause of implant failure and revision surgery [72]. Metallic wear particles and corrosion products trigger inflammatory cascades, osteolysis, and systemic metallic ion accumulation [73]. Predicting degradation trajectories prospectively, before device failure manifests clinically, is therefore a high-priority application for ML in orthopedics.

### 7.1. Corrosion Classification

A systematic review by Kurtz et al. surveyed AI/ML corrosion work on physiologically relevant damage modes: fretting, pitting, and crevice corrosion [74]. Of the fifty reviewed articles, only seven involved orthopedic biomaterials directly, underscoring how far this domain lags behind other engineering fields. Fretting corrosion at femoral tapers had the most developed ML literature, including studies predicting Goldberg damage scores from retrieval data. It outlined orthopedic-adaptable designs: image-based classification of pitting and crevice damage and prediction of corrosion-promoting conditions, pointing to a largely unexplored agenda. Codirenzi et al. trained a CNN on 725 retrieved modular femoral stems to classify Goldberg scores, reaching 98.3% accuracy distinguishing no/mild from moderate/severe corrosion, demonstrating ML as a high-throughput screening tool for retrieval programs [22].

### 7.2. Wear and Failure Prediction

Wear prediction in total knee replacement (TKR) has advanced markedly. Perrone et al. trained a transformer-CNN encoder-decoder to predict linear wear distribution on polyethylene liners from 314 gait time-series (ISO 14243-3 loading), using a validated FE model as ground truth [47]. The surrogate cut computation from days to minutes, with mean absolute percentage errors below 6% for wear-scar geometry and SSIM and NMI above 0.88. With wearable gait sensors, it could enable patient-specific wear prediction from real-world activity without bespoke FE models. Borjali et al. earlier built data-driven models for polyethylene wear in hip bearings from pin-on-disc data, predicting wear rate for new configurations and quantifying the contributions of contact stress, sliding distance, and cross-shear: design-of-experiments guidance that could reduce costly tribological testing [48].

Masciulli et al. advanced imaging-based prediction of implant failure with a combined CNN and recurrent neural network predicting hip prosthesis failure from sequences of two or three longitudinal anteroposterior radiographs [49]. Using GRU or LSTM recurrent layers and pretrained DenseNet CNNs, the three-image model reached a positive predictive value of 0.966 and F1 of 0.933 on validation. On external validation (14 patients), the model reached an accuracy of 0.786. Capturing temporal and spatial evolution rather than a single time point, this approach is a step toward automated clinical surveillance of implant status.

Ampadi Ramachandran et al. explored continuous post-surgical monitoring using tribocorrosion and acoustic emission data with ML classification in an in vitro case study [21]. Their non-invasive AI hip monitoring system aims to detect the onset of irreversible material change during daily activity, shifting implant management from reactive revision toward preventive intervention. Mutu integrated FE analysis with ML (Decision Tree, Multilayer Perceptron, Support Vector Machine) to predict stress and displacement in plate-screw fixation of femoral-shaft fractures across three gap sizes, two materials (Ti-6Al-4V and 316L stainless steel), and axial loads from 400 to 1200 N [50]. MLP and SVM substantially outperformed Decision Trees, and Ti-6Al-4V consistently showed lower von Mises stresses and displacements than stainless steel, validating the clinical preference for titanium in load-bearing fixation.

### 7.3. Cross-Domain Integration

The integration of corrosion and wear prediction with upstream alloy and surface design represents an important but largely unrealized opportunity. Current ML models in this domain are developed in isolation from the alloy composition and surface engineering literature reviewed in preceding sections, yet in-service degradation performance is intimately determined by both bulk composition and surface state. Multi-domain ML frameworks that propagate uncertainty from alloy design through surface modification to in-service degradation prediction would provide substantially more clinically useful tools than any single-domain model.

Two wear studies here (Perrone et al. [47] and Borjali et al. [48]) concern ultra-high-molecular-weight polyethylene rather than the metal directly; they are included because metal-on-polyethylene wear drives the osteolysis and revision that metallic-implant design seeks to mitigate. Current models also treat corrosion and wear as separable, whereas in vivo degradation is dominated by tribocorrosion, where mechanical and electrochemical damage couple. Mechanistic models will need contact stress and fretting amplitude, oxide-film stability and repassivation kinetics, body-fluid chemistry and pH, protein adsorption, and third-body effects, variables largely absent from the studies reviewed here.

### 7.4. Critique

This domain is the least mature: of fifty corrosion studies in one systematic review, only seven addressed orthopedic biomaterials [74], and most models are retrospective classifiers trained on retrieval or finite-element data. The most instructive cautionary example is the longitudinal radiographic failure-prediction model, which reached a positive predictive value of 0.966 on internal validation but fell to an accuracy of only 0.786 on an external cohort of fourteen patients [49]. Reporting both internal and external metrics, as that study did, should be the norm rather than the exception.

## 8. Discussion

Synthesizing across the five domains reviewed, several cross-cutting themes emerge that both characterize the current state of the field and define the priorities for future progress.

First, the trajectory of ML sophistication is clearly upward. Early applications consisted primarily of regression models trained on small, single-property experimental datasets, which were valuable for hypothesis generation but limited in scope and transferability [13,16]. The more recent literature features deep neural networks, transformer architectures, Bayesian active learning, multi-agent generative AI, physics-informed hybrid models, and GAN-based data augmentation strategies that integrate data-driven prediction with first-principles constraints [75,76]. In metallic orthopedic implants specifically, this evolution is visible in the progression from single-composition regression through multi-property DFT-validated screening to fully autonomous multi-agent alloy design platforms and GAN-PPO composition generation [18,23,24,27].

Second, data remains the dominant bottleneck across all domains [12,77]. Experimental databases for alloy composition, AM process–property relationships, biological surface responses, and in vivo tribocorrosion outcomes are all small, heterogeneous, and often not widely available [74,78,79,80]. This drives reliance on transfer learning, first-principles validation, physics-informed regularization, GAN-based data augmentation, and active learning strategies that extract maximum information per experiment [76,77,81]. However, these strategies complement rather than substitute for expanded data generation. Coordinated efforts to build open, standardized databases, with agreed measurement protocols, metadata standards, and data-sharing agreements, are arguably the highest-leverage infrastructure investment the field could make.

Third, model interpretability is a critical and partially unresolved challenge. In safety-critical medical device applications, predictions that cannot be mechanistically explained are unlikely to satisfy regulatory requirements or clinical trust thresholds. Studies incorporating interpretability tools, including SHAP analysis [38], Grad-CAM [31], feature importance rankings, and dropout uncertainty estimation [29], represent best practice, providing physically meaningful insights alongside numerical predictions [82]. The development of interpretability frameworks specifically calibrated for materials design tasks, in which physical laws constrain the admissible solution space, remains an active and important research direction.

Interpretability requirements are also architecture-specific. For tree-based and random forest models, feature-importance and SHAP analyses give direct, physically auditable attributions suited to the tabular descriptors of alloy design [38]. For convolutional networks on micrographs or radiographs, saliency methods such as Grad-CAM give spatial but only qualitative explanations, as in the MPR-Net melt-pool maps [31]. For physics-informed networks, interpretability comes less from post hoc attribution than from the physical residuals in the loss function, which can be inspected to confirm predictions respect conservation laws. Distinct from general healthcare ML, explanations here must agree with known physical constraints, including monotonic property–composition trends, phase-stability rules, and defect–fatigue mechanisms, so interpretability acts as a physical-plausibility check, not merely a transparency aid.

Beyond the architecture-specific considerations above, explainable artificial intelligence (XAI) is likely to be a prerequisite for regulatory approval and clinical adoption rather than an optional enhancement. Regulators and clinicians are unlikely to accept a black-box recommendation that a given alloy, lattice, or surface is safe without a mechanistic account of why the model reached that conclusion, and emerging regulatory guidance for AI-enabled medical devices increasingly expects transparency and traceability of model reasoning [83,84,85]. Post-hoc, model-agnostic techniques are especially valuable in this setting: SHAP (SHapley Additive exPlanations) apportions a prediction to its input features using a game-theoretic framework [82], allowing designers to confirm, for example, that a predicted reduction in elastic modulus is driven by physically sensible descriptors such as β-stabilizer content rather than spurious dataset artifacts, whereas LIME (Local Interpretable Model-agnostic Explanations) fits a simple surrogate around an individual prediction to expose the features responsible for that specific case [82]. Applied consistently, and cross-checked against established metallurgical and mechanobiological mechanisms, such tools convert opaque predictions into auditable design rationales, support the risk documentation that regulatory submissions require [83,84,85], and help build the clinical trust required for ML-designed implants to progress from proof-of-concept toward approval.

Two fundamentally different ML task classes, often conflated in the literature, warrant distinction. Generative models (e.g., GANs for alloy or lattice exploration) propose candidates and are judged by the experimental validity, novelty, and manufacturability of their outputs; their main risks, namely extrapolation into unrealizable regions and training-set bias, demand downstream confirmation and explicit novelty and constraint-satisfaction reporting [75]. Surrogate models (e.g., networks emulating finite-element or CALPHAD computations) approximate a well-defined mapping and are judged by accuracy and uncertainty relative to the solver they replace, with attention to extrapolation beyond the sampled space [76]. Conflating them, treating a surrogate’s agreement with its training simulator as experimental validation, overstates evidential strength; the two classes need distinct validation criteria.

Several reliability concerns cut across these tasks and deserve more consistent reporting than the reviewed literature provides. Small and simulation-generated datasets invite overfitting and data leakage, particularly where near-duplicate compositions, specimens from a single build, or augmented samples are split across training and test partitions [24,45]; learning curves, nested cross-validation, and explicit leakage audits are rarely reported. Transferability across alloy systems, AM machines, and laboratories is seldom tested, yet is precisely the property required for clinical deployment. And models trained on simulated data inherit the assumptions and idealizations of the underlying solver [34,39,47], so their apparent accuracy bounds the simulator’s fidelity rather than physical reality.

Fourth, external validation against independent large-scale clinical datasets remains strikingly rare. Nearly all reviewed studies validated ML models against held-out portions of the same experimental dataset used for training, or at most against a second independent experiment conducted in the same laboratory. The study by Masciulli et al. represents a partial exception, employing an external validation cohort of 14 patients, though this remains modest by clinical standards [49]. No study in this review validated predictions against a national or international implant registry outcome dataset. Bridging this gap requires prospective study designs, data linkage between manufacturing records and registry databases, and sustained interdisciplinary collaboration between computational materials scientists and clinical outcome researchers.

A consistent vocabulary for validation would clarify the evidentiary status of these models. We propose a three-tier validation framework distinguishing computational validation, in which predictions are checked against held-out simulation or first-principles data; experimental validation, in which predictions are confirmed by independent physical fabrication and testing or by retrieval analysis; and clinical validation, in which predictions are tested against in vivo, prospective, or registry-level patient outcomes. Computational validation predominates in the alloy-discovery and lattice domains, while experimental validation is most common overall; clinical validation is reached by only one study. Computationally validated models should not be presented as equivalent to experimentally or clinically supported ones; each study’s tier is recorded in Table 1.

A recurring obstacle across all five domains is the absence of shared minimum reporting standards, which limits reproducibility, data sharing, and cross-study comparability [77,78]. We therefore propose that future studies in this field adopt a minimum data-reporting framework specifying, at a minimum: (i) full dataset provenance and size, including the number of unique compositions, specimens, or images and whether they are of experimental or simulated origin; (ii) the material system and processing conditions in sufficient detail to permit replication; (iii) a complete description of model architecture, hyperparameters, feature set, and training procedure; (iv) the data partitioning and validation strategy, with explicit reporting of the measures taken to prevent data leakage between training and test sets, such as grouping near-duplicate compositions or specimens originating from a single build; (v) uncertainty quantification accompanying each prediction rather than point estimates alone; (vi) the validation tier reached, distinguishing computational, experimental, and clinical evidence; and (vii) deposition of data and code in open, versioned repositories under FAIR (Findable, Accessible, Interoperable, Reusable) principles wherever proprietary constraints allow [55,78]. Widespread adoption of such a checklist, ideally coordinated through journals and professional societies, would materially improve reproducibility and enable the pooled, cross-study datasets on which the next generation of integrated, multi-domain models will depend [55,77].

Fifth, the regulatory landscape for AI-designed medical devices is nascent and evolving. While the US Food and Drug Administration has issued guidance on AI/ML-based software as a medical device and proposed frameworks for predetermined change control plans, no specific regulatory pathway yet exists for implants whose composition, architecture, or surface properties were primarily determined by ML optimization [83,84,85]. Engagement between the research community, regulatory agencies, and standards bodies to develop fit-for-purpose validation and approval frameworks is an urgent priority.

A fit-for-purpose regulatory pathway for ML-designed metallic implants will need to be technical and specific rather than generic. At minimum it should encompass traceable and version-controlled training datasets, predetermined and controlled model-update protocols, quantitative uncertainty reporting accompanying every prediction, qualification of the additive-manufacturing process itself, and the full suite of conventional device evidence, namely fatigue testing, corrosion and tribocorrosion testing, and biocompatibility assessment, applied to the ML-derived design rather than waived on the basis of computational prediction [83,84,85]. Post-market surveillance, ideally linked to registries, is essential to detect the out-of-distribution failures that internal validation cannot anticipate.

The five domains sit at markedly different levels of methodological maturity, from the relatively advanced alloy-discovery and additive-manufacturing literatures to the still-nascent corrosion and wear domain. Table 2 compares the dominant model types, dataset sizes, and validation strategies across domains, and Table 3 summarizes their comparative maturity and the highest validation tier reached in each.

## 9. Future Directions

**Figure 2:**
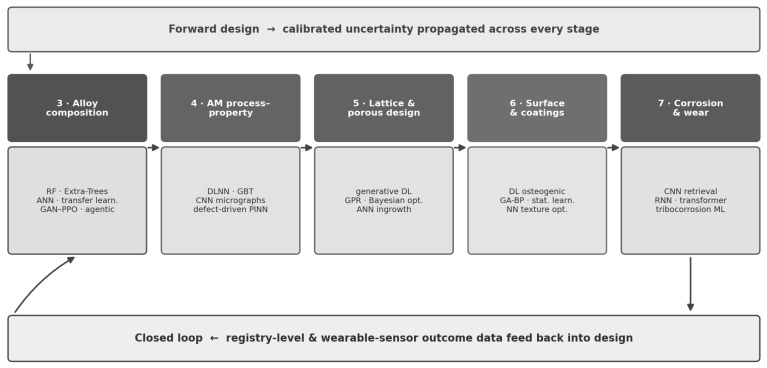
Conceptual integrated, multi-scale ML pipeline spanning the five development stages, with calibrated uncertainty propagated forward and clinical outcome data fed back into design.

The most transformative near-term opportunity is integrated, multi-scale, multi-domain ML pipelines spanning alloy composition through in-service degradation. Such pipelines would connect alloy composition prediction (Section 3) through AM process optimization (Section 4) and structural design (Section 5) to surface engineering (Section 6) and corrosion and wear prediction (Section 7), propagating uncertainties across each stage and optimizing across the full performance envelope simultaneously. Initial steps toward such integration are visible in the work of Zaheer et al., who connected AM process parameters through mechanical properties to biomechanical healing simulation, and Su et al., who designed alloys with simultaneous consideration of AM processability and biological performance, but full end-to-end pipeline integration remains an open research challenge (Figure 2) [17,34].

Realizing such pipelines faces concrete and underappreciated technical barriers. The five domains currently use incompatible data representations, composition vectors, voxelized or mesh-based geometries, image stacks, and time-series signals, recorded at different length and time scales and in inconsistent units, with no shared schema or metadata standard. Propagating calibrated uncertainty across these heterogeneous stages, rather than reporting it in isolation at each step, is itself an unsolved problem, as is reconciling the differing fidelity of the simulators that feed each stage. Progress will require agreed data standards and ontologies for the implant-development pipeline [55,77,78] before genuine end-to-end optimization becomes feasible.

The advent of agentic AI systems, exemplified by AtomAgents, suggests that orchestration of multi-domain pipelines may itself be automatable, with AI agents autonomously deciding which computational tools to invoke, interpreting results, and designing the next experiment [27]. This closed-loop, self-directed materials discovery vision is technically plausible with current AI capabilities but requires governance structures to ensure that autonomous systems respect safety constraints, produce reproducible outputs, and generate interpretable audit trails compatible with regulatory requirements.

This optimism must be tempered: AtomAgents and comparable agentic platforms have so far been demonstrated only on computational alloy design benchmarks and have not been validated in any real-world orthopedic implant scenario. Their safety, reproducibility, and failure modes under autonomous operation are essentially uncharacterized, and in a safety-critical medical device context such systems should at present be regarded as exploratory research tools rather than design engines whose outputs can be trusted without exhaustive independent verification.

The incorporation of patient-specific data, including genomic, biomechanical, radiographic, and activity-based information, into implant design workflows represents another frontier with substantial clinical potential. ML models that predict how a given patient’s bone quality, gait characteristics, and metabolic profile will interact with a specific implant design could enable truly individualized device selection and fabrication. Wearable sensor-based continuous monitoring systems, as envisioned by Ampadi Ramachandran et al., could close the loop by feeding real-world in-service performance data back into design optimization databases, creating a virtuous cycle of learning and improvement [21].

Finally, prospective clinical validation studies, in which implants designed with ML assistance are compared against conventional designs in randomized or registry-linked cohort studies, are essential to establish whether computational predictions of improved performance translate to better patient outcomes. The field currently possesses considerable evidence that ML can optimize individual material properties in silico and in vitro; it largely lacks evidence that these optimizations improve implant survivorship, patient-reported outcome measures, or revision rates. Designing and executing such studies should be a strategic priority for the next decade.

## 10. Conclusions

Across the five development stages surveyed, the value of machine learning in metallic orthopedic implants lies less in any single predictive result than in its emerging ability to connect alloy chemistry, manufacturing, architecture, surface, and degradation within a common optimization framework. Realizing clinical benefit depends on overcoming shared barriers: small, heterogeneous datasets, mostly internal validation, limited interpretability, and the absence of fit-for-purpose regulation, through open standardized databases, prospective registry-linked validation, and coordinated engagement among researchers, industry, and regulators.

## Figures and Tables

**Table 1:** Characteristics of the 33 primary studies included in the review, grouped by development domain. Validation tier: Comp. = computational (simulation/first-principles); Exp. = experimental (fabrication, retrieval, in vitro or in vivo); Clin. = clinical (in vivo/registry/patient outcomes). n = reported dataset size; n/r = not reported; n/a = not applicable.

No.	Study	Domain	Material System	ML/AI Method	n	Validation	Key Result/Limitation
1	Marković et al. 2023 [23]	Alloy	Ti alloys (β-type)	Extra-Tree regression	246	Comp.	Predicts Young’s modulus (specific heat a top descriptor); Monte Carlo screen of ~10^7^ compositions flags Ti-Zr-Sn-Mn-Nb < 70 GPa. In silico only.
2	Salvador et al. 2020 [18]	Alloy	Ti-Nb-Zr	LR/RF/ANN + DFT	1800+	Comp. (DFT)	RF within ~4 GPa of DFT; ~22 at.% Zr lowers modulus, Nb > 14.8 at.% suppresses ω-phase. No fabrication.
3	Su et al. 2026 [17]	Alloy	Ti-Nb-Ta-Zr-Sn (LPBF)	Multi-property ML framework	n/r	Exp.	Fabricated alloy reaches E ≈ 42.7 GPa with ~30.9% ductility; strongest validation in this domain.
4	Zhang et al. 2026 [24]	Alloy	Multicomponent alloys	GAN + PPO reinforcement	synthetic	Comp.	Efficient inverse design from sparse data; synthetic samples may inherit seed data bias.
5	Gupta et al. 2021/23 [25,26]	Alloy	High-entropy alloys	Cross-property deep transfer learning	large → small	Comp.	Predicts up to 41 properties from composition; mitigates sparse HEA data.
6	Ghafarollahi and Buehler 2025 [27]	Alloy	Multiple alloy systems	Multi-agent LLM (AtomAgents)	n/a	Comp.	Autonomous physics-aware design; demonstrated only on computational benchmarks.
7	Hassanin et al. 2021 [28]	AM	Ti-6Al-2Sn-4Zr-6Mo	Deep-learning NN	small	Exp.	~3% porosity/0.2% hardness error; identifies 77–113 J/mm^3^ densification window.
8	Moon et al. 2021 [29]	AM	Ti-6Al-4V	Dropout NN (uncertainty)	197	Exp.	Predicts fatigue life (log N) with uncertainty; pore size/area most predictive.
9	Hossain et al. 2026 [30]	AM	Ti-6Al-4V (LPBF)	Multimodal surrogate + GBT	small	Exp.	YS/UTS R^2^ ≈ 0.87–0.90 but surface roughness/hardness R^2^ ≈ 0.11–0.12 (explicit failure mode).
10	Zhang et al. 2024 [31]	AM	316L stainless steel	CNN (MPR-Net)	images	Exp.	Tensile R^2^ = 0.96, hardness R^2^ = 0.91; Grad-CAM localizes melt pool regions.
11	Pan et al. 2024 [32]	AM	Ti-6Al-4V	DeepLabV3+ segmentation	CT data	Exp.	98.2% defect-pixel accuracy; fatigue-life error within ±2.2× scatter band.
12	Wang et al. 2023 [33]	AM	Multiple AM systems	Defect-driven PINN	small	Comp./Exp.	Outperforms data-driven and fracture-mechanics PINN in cross-dataset generalization.
13	Zaheer et al. 2025 [34]	AM	Ti-6Al-4V (SLM)	AI regression on FE	FE set	Comp. (FE)	Real-time bone healing prediction; a surrogate of FE rather than experiment.
14	Sanchez et al. 2021 [35]	AM	Ni-base Alloy 718	Ensemble + feature importance	n/r	Exp.	Creep-rate prediction (best error 1.40%); density, porosity, orientation dominate.
15	Minku and Ghosh 2025 [36]	Lattice	Ti diamond lattice (TAR)	ML on FE dataset	FE set	Comp. (FE)	Intermediate ~70% porosity optimal; 80% detrimental (informative negative result).
16	Minku and Ghosh 2026 [19]	Lattice	Ti rhombic dodecahedron	ANN (4 algorithms)	FE set	Comp. (FE)	Identifies design maximizing bone formation while raising von Mises stress.
17	Lee et al. 2022 [37]	Lattice	Metallic lattices	Deep learning + NN-GO	n/r	Exp.	Joint-region material redistribution raises modulus/strength; LPBF-validated.
18	Gongora et al. 2024 [38]	Lattice	Lattice structures	ML surrogate + SHAP + Bayesian opt.	n/r	Comp.	R^2^ > 0.95 with ~5× fewer FE simulations; SHAP gives interpretable drivers.
19	Judawisastra et al. 2025 [39]	Lattice	Gyroid (TPMS)	Gaussian process regression (GPR)	10–75% ρ	Exp.	Outperforms Gibson–Ashby at high relative density.
20	Shetty et al. 2025 [40]	Lattice	PLA+ scaffolds (non-metallic)	Back-propagation ANN	wall 1–2 mm	Exp.	Displacement R^2^ = 0.9991; included for transferable TPMS/surrogate methodology.
21	Jana et al. 2025 [41]	Lattice	Ti/Mg scaffold	AI on homogenization data	18 geom.	Comp.	Predicts Ti/Mg ratio giving time-evolving stiffness for degradable scaffolds.
22	Li et al. 2025 [20]	Surface	Ti & alloy surfaces	Deep learning (OIODNet)	images	Exp. (in vitro)	>0.98 across six metrics; validated against ALP for metal-polyphenol coatings.
23	Lu et al. 2022 [42]	Surface	Ti + Ag nanoparticles	GA-BP neural network	256	Exp. (in vitro)	<10% error; balances antibacterial efficacy against cytocompatibility.
24	Ghosh et al. 2021 [43]	Surface	Implant macro-textures	BPNN + FE + GA	FE set	Comp. (FE)	Optimized rib macro-textures yield uniform bone-implant interface stress.
25	Fang et al. 2023 [44]	Surface	Ternary functional surface	Statistical learning	gradient	Exp. (in vivo)	Screens osteo/angio/neurogenic response; in vitro and in vivo validated.
26	Barai et al. 2026 [45]	Surface	Mg-alloy coating	RSM + ANN + multi-objective GA	augmented	Exp. (in vitro)	R^2^ > 0.99; superhydrophobic (152°), 92.4% corrosion resistance.
27	Khakbiz and Esteghza 2025 [46]	Surface	PEO-coated Mg-Zn-Si	Gaussian process regression	n/r	Exp.	Predicts surface topography; hardness raised from 81 to 480 HV.
28	Codirenzi et al. 2023 [22]	Corrosion	CoCr femoral stems	CNN	725	Exp. (retrieval)	98.3% accuracy classifying none/mild vs. moderate/severe corrosion.
29	Perrone et al. 2025 [47]	Wear	UHMWPE liner (bearing)	Transformer-CNN surrogate	314	Comp. (FE)	Wear MAPE < 6%, minutes vs. days; metal-on-PE wear relevant to revision.
30	Borjali et al. 2019 [48]	Wear	UHMWPE (hip)	Data-driven ML	pin-on-disc	Exp.	Predicts wear rate and ranks parameter contributions; metal-on-PE articulation.
31	Masciulli et al. 2025 [49]	Corrosion	Hip-prosthesis radiographs	CNN + RNN (GRU/LSTM)	longitudinal	Clin. (external)	PPV 0.966 internal falls to 0.786 accuracy on external cohort of 14 patients.
32	Ampadi Ramachandran et al. 2023 [21]	Corrosion	Hip implant (tribocorrosion)	ML classification + acoustic emission	in vitro	Exp. (in vitro)	Proof-of-concept non-invasive degradation monitoring.
33	Mutu 2026 [50]	Corrosion	Ti-6Al-4V/316L plate-screw	DT/MLP/SVM + FE	3 gaps	Comp. (FE)	MLP and SVM outperform DT; Ti shows lower von Mises stress (loads 400–1200 N).

Abbreviations: ANN = artificial neural network; CNN = convolutional neural network; DFT = density functional theory; FE = finite element; GA = genetic algorithm; GAN = generative adversarial network; GBT = gradient-boosted tree; GPR = Gaussian process regression; GRU = gated recurrent unit; LSTM = long short-term memory; MLP = multilayer perceptron; PINN = physics-informed neural network; PPO = proximal policy optimization; RSM = response surface methodology; SHAP = Shapley additive explanations; SVM = support vector machine; TPMS = triply periodic minimal surface.

**Table 2:** Comparison of dominant machine learning methods, typical dataset sizes, validation strategies, and clinical relevance across the five development domains.

Domain	Dominant ML Methods	Typical Dataset Size	Primary Validation Strategy	Clinical Relevance
Alloy composition discovery	Tree ensembles, ANN, transfer learning, GANs, agentic LLMs	Hundreds to low thousands (often public databases)	Held-out data and DFT/first-principles agreement	Lower-modulus, biocompatible alloys to reduce stress shielding
AM process–property optimization	Deep NNs, gradient boosting, CNNs on images, physics-informed NNs	Tens to a few hundred specimens	Held-out specimens; some printed validation	Defect- and fatigue-controlled load bearing parts
Lattice and porous design	Generative DL, Gaussian process regression, Bayesian optimization, ANN	Simulation-generated; few printed samples	FE ground truth; limited fabrication	Tuned stiffness and porosity for osseointegration
Surface engineering and coatings	Deep learning, GA-BP NN, statistical learning, NN optimization	Hundreds of in vitro data points	In vitro assays; rare in vivo confirmation	Osteogenic, antibacterial, corrosion-resistant surfaces
Corrosion and wear prediction	CNN, RNN, transformer surrogates, classical classifiers	Tens to hundreds (retrieval/FE)	Mostly internal; one external clinical cohort	Longevity, tribocorrosion and failure prediction

**Table 3:** Comparative methodological maturity and highest validation tier reached in each domain, with the principal outstanding gap.

Domain	Methodological Maturity	Highest Validation Tier Reached	Key Remaining Gap
Alloy composition discovery	High (regression → physics-constrained and generative)	Experimental (fabricated low-modulus alloys)	Independent external and biological validation of generated candidates
AM process–property optimization	High (data-driven, physics-informed and surrogate paradigms)	Experimental (printed and tested specimens)	Cross-machine/cross-lab transferability of fatigue prediction
Lattice and porous design	Moderate (mature surrogates on simulated data)	Mostly computational; limited fabrication	Validation against in vivo bone ingrowth, not FE surrogates
Surface engineering and coatings	Moderate (strong in vitro accuracy)	Experimental, predominantly in vitro	Datasets linking surface metrics to in vivo and registry outcomes
Corrosion & wear prediction	Low (least mature; small retrospective sets)	Clinical but only single external cohort	Prospective, externally validated tribocorrosion models

## Data Availability

No new data were created or analyzed in this study. Data sharing is not applicable to this article.
